# 3D Genome Constrains Breakpoints of Inversions That Can Act as Barriers to Gene Flow in the Stickleback

**DOI:** 10.1111/mec.17814

**Published:** 2025-05-31

**Authors:** Yo Y. Yamasaki, Atsushi Toyoda, Mitsutaka Kadota, Shigehiro Kuraku, Jun Kitano

**Affiliations:** ^1^ Ecological Genetics Laboratory National Institute of Genetics Mishima Shizuoka Japan; ^2^ Genetics Course The Graduate University for Advanced Studies Mishima Shizuoka Japan; ^3^ Comparative Genomics Laboratory National Institute of Genetics Mishima Shizuoka Japan; ^4^ Laboratory for Phyloinformatics RIKEN Center for Biosystems Dynamics Research (BDR) Kobe Hyogo Japan; ^5^ Laboratory for Developmental Genome System RIKEN Center for Biosystems Dynamics Research (BDR) Kobe Hyogo Japan; ^6^ Molecular Life History Laboratory Department of Genomics and Evolutionary Biology, National Institute of Genetics Mishima Shizuoka Japan

**Keywords:** 3D genome, genome assembly, Hi‐C, introgression, mutation bias, sticklebacks

## Abstract

DNA within the nucleus is organised into a well‐regulated three‐dimensional (3D) structure. However, how such 3D genome structures influence speciation processes remains largely elusive. Recent studies have shown that 3D genome structures influence mutation rates, including the occurrence of chromosomal rearrangement. For example, breakpoints of chromosomal rearrangements tend to be located at topologically associating domain (TAD) boundaries. Here, we hypothesised that TAD structures may constrain the location of chromosomal inversions and thereby shape the genomic landscape of divergence between species with ongoing gene flow, given that inversions can act as barriers to gene flow. To test this hypothesis, we used a pair of Japanese stickleback species, *Gasterosteus nipponicus* (Japan Sea stickleback) and *G. aculeatus* (three‐spined stickleback). We first constructed chromosome‐scale genome assemblies of both species using high fidelity long reads and high‐resolution proximity ligation data and identified several chromosomal inversions. Second, via population genomic analyses, we revealed higher genetic differentiation in inverted regions than in colinear regions and no gene flow within inversions, which contrasts with the significant gene flow in colinear regions. Third, using Hi‐C data, we revealed 3D genome structures of sticklebacks, delineated by A/B compartments and TADs. Finally, we found that inversion breakpoints tend to be located at TAD boundaries. Thus, our study demonstrates that the 3D genome constrains breakpoints of inversions that can act as barriers to gene flow in the stickleback. Further integration of 3D genome analyses with population genomics could provide novel insights into how the 3D genome influences speciation.

## 1 Introduction

DNA within the nucleus is organised into a well‐regulated three‐dimensional (3D) structure (Oudelaar and Higgs 2021; Szabo et al. 2019; Zheng and Xie 2019). For example, the genome is spatially segregated into A and B compartments (Lieberman‐Aiden et al. 2009; Rao et al. 2014; Wang et al. 2016). The A compartments contain actively expressed genes with low chromatin density, making them more accessible to RNA polymerase and other proteins. By contrast, the B compartments are suppressed regions and are tightly condensed, reducing protein accessibility. Additionally, there are smaller local physically interacting domains, known as topologically associating domains (TADs) (Dixon et al. 2012; Nora et al. 2012). Genes within a TAD often share the same *cis*‐regulatory regions and exhibit coordinated gene expression (Bonev et al. 2017; Dixon et al. 2016; Nora et al. 2012; Ramírez et al. 2018; Symmons et al. 2014). Therefore, changes in 3D genome structures can cause not only diseases (Bonev and Cavalli 2016; Spielmann et al. 2018) but also phenotypic evolution (Marlétaz et al. 2023). Furthermore, 3D genome structures can influence mutation rates across the genome, with TAD boundaries tending to have higher mutation rates (Canela et al. 2017). Despite this accumulating knowledge on 3D genome structures, we know little about any roles that they might play in speciation.

Chromosomal structural changes potentially contribute to genomic divergence and speciation (Noor et al. 2001; Rieseberg 2001; Berdan et al. 2024). Chromosomal structural differences can cause not only meiotic arrest and the production of aneuploid gametes in heterokaryotypes, but also recombination suppression at the rearranged regions (Berdan et al. 2024; Reifová et al. 2023). For example, recombination is often suppressed in inverted regions, and thus these regions may accumulate or maintain alleles important for local adaptation and reproductive isolation (Felsenstein 1981; Kirkpatrick and Barton 2006; Yeaman 2013) and act as barriers to gene flow between hybridising populations (Faria et al. 2019; Navarro and Barton 2003; Noor et al. 2001; Rieseberg 2001; Wellenreuther and Bernatchez 2018). Additionally, inversions themselves may confer direct fitness advantages for local adaptation (Villoutreix et al. 2021). By reducing gene flow, genetic differentiation at the inverted regions may be elevated compared with that at the colinear regions (Feder and Nosil 2009; Guerrero et al. 2012; Rieseberg 2001). Consistent with this idea, several empirical studies have shown that inverted regions overlap with genomic islands of divergence between populations (Harringmeyer and Hoekstra 2022; Huang et al. 2020; Jones et al. 2012; Matschiner et al. 2022; McGaugh and Noor 2012; Michel et al. 2010; Le Moan et al. 2024; Nosil et al. 2023; Todesco et al. 2020), although other studies have found no evidence for elevated genetic divergence in the inversions compared with the level in the colinear regions (Davey et al. 2017; Lucek et al. 2019). Thus, chromosomal inversions can contribute to local adaptation and possibly speciation.

3D genome structures can determine where chromosomal inversions tend to occur, and therefore these structures may influence genomic patterns of genetic differentiation and introgression between hybridising species. Breakpoints of chromosomal rearrangements, including inversions, often overlap with TAD boundaries in diverse taxa, including both vertebrates (Choudhary et al. 2023; Fudenberg and Pollard 2019; Krefting et al. 2018; Lazar et al. 2018; Li et al. 2022; Okhovat et al. 2023; Yang et al. 2020; Yin et al. 2021) and invertebrates (Liao et al. 2021; Lukyanchikova et al. 2022; Torosin et al. 2020; Wright and Schaeffer 2022; Zhou et al. 2024). There are several explanations for these correlations. First, TAD boundaries are vulnerable to double‐strand DNA breaks (Canela et al. 2017), which can elevate the frequency of rearrangements at such boundaries (Lagerstedt 1997; So et al. 2017). Second, chromosomal rearrangements spanning TAD boundaries are so deleterious that purifying selection acts to maintain coordinated gene expression within the TAD (Fudenberg and Pollard 2019; Krefting et al. 2018; Lazar et al. 2018; Liao et al. 2021). Indeed, it has been reported that chromosomal mutations that alter TADs are often pathogenic in humans (Spielmann et al. 2018). Either way, we hypothesised that TAD structures may constrain the location of chromosomal inversions and indirectly influence the genomic landscape of divergence between hybridising populations or species. Although studies comparing TAD structures among species have been performed, the majority of them investigated distantly related species that do not hybridise (Brideau et al. 2006; Liao et al. 2021; Lukyanchikova et al. 2022; Thybert et al. 2018; Torosin et al. 2020). As such, it has remained unclear whether TADs constrain the breakpoints of inversions that contribute to local adaptation or reproductive isolation (Berdan et al. 2024; Mohan et al. 2024).

The sympatric Japanese species pair of *Gasterosteus nipponicus* (Japan Sea stickleback) and *G. aculeatus* (three‐spined stickleback) is an excellent model system to investigate the relationships among structural variation, 3D genome structure, and introgression. It is estimated that these two species started to diverge approximately 0.68–1 million years ago, but they have continued to exchange genes at a low rate since then (Ravinet et al. 2018). For example, these two species are currently sympatric in eastern Hokkaido, where ongoing gene flow is observed (Ravinet et al. 2018, 2021). Genomic divergence is high across the genome, with small localised regions of introgression (Ravinet et al. 2018, 2021). *G. nipponicus* has a neo‐sex chromosome system due to fusion between an ancestral Y chromosome and a previously autosomal chromosome IX (chrIX) (Kitano et al. 2009). Previous studies have shown that genetic differentiation and introgression tend to be high and low, respectively, at neo‐sex chromosomes compared with the levels at autosomes (Yoshida et al. 2014; Ravinet et al. 2018), indicating that sex chromosome fusions can act as barriers to gene flow. However, we do not know whether these species have any chromosomal inversions on their autosomes, whether the inversions act as barriers to gene flow, or whether inversion breakpoints are constrained by TAD structures.

Against this background, the goal of the present study is to investigate the relationship among 3D genome structure, inversions, and introgression in this pair of sympatric Japanese species. To achieve this goal, we first constructed high‐quality genome assemblies for *G. nipponicus* and Japanese *G. aculeatus* using PacBio HiFi long‐read sequencing and Dovetail Omni‐C scaffolding technologies. Next, using these genome assemblies, we identified several inversions between these two species and tested whether these inversions act as barriers to gene flow. Then, we conducted high‐coverage Hi‐C analysis to investigate their 3D genome structures. Finally, we examined whether the inversions occur in association with TAD structures.

## 2 Materials and Methods

### 2.1 De Novo Haplotype‐Phased Genome Assembly

Using PacBio HiFi sequencing and Hi‐C, we constructed a haplotype‐phased de novo assembly of *G. nipponicus* and a Japanese Pacific Ocean lineage of *G. aculeatus* . For *G. nipponicus*, one wild‐caught male collected from a tidepool near Biwase River, Hamanaka, Hokkaido, Japan, was used (Kume and Mori 2009). For *G. aculeatus* , one male derived from a pure cross of anadromous stickleback collected in Bekanbeushi River, Akkeshi, Hokkaido, Japan, was used (Kitano et al. 2009). Fish collection was conducted under the permit issued by Hokkaido Prefecture (2018‐6, 2022‐13, and 2024‐19). All animal experiments were approved by the Institutional Animal Care and Use Committee of the National Institute of Genetics (R5‐19 and R6‐18).

For the PacBio sequencing, high‐molecular‐weight DNA was isolated from blood with Smart DNA Prep (m) (IST Innuscreen GmbH, Berlin, Germany). Extracted DNA was sheared using a g‐tube (Covaris, MA, USA) for *G. nipponicus*, and using Megaruptor3 (Diagenode, Seraing, Belgium) for *G. aculeatus* . Target‐size DNA was extracted using Sage ELF (Sage Science, MA, USA). Libraries were constructed using SMRTbell Prep Kit 3.0 and sequenced in HiFi mode using one Sequel II SMRT Cell 8M of PacBio Sequel II for *G. nipponicus* and of PacBio Sequel IIe for *G. aculeatus* . HiFi reads were generated by *DeepConsensus* v1.2 (Baid et al. 2023). Finally, we obtained 45,003,840,704 bp for *G. nipponicus* and 38,272,284,826 bp for *G. aculeatus* of the PacBio HiFi reads respectively.

To construct haplotype‐phased contig assembly and scaffolding, we conducted Hi‐C using the same individuals as used for the HiFi sequencing. Dovetail Omni‐C kit (Cantata Bio, CA, USA) was used to construct the libraries. Trunk muscle tissue was fixed using formaldehyde, and the nuclease enzyme mix was used for digesting crosslinked DNA. Obtained Omni‐C libraries were sequenced on NovaSeq 6000 (Illumina, CA, USA) in the 150 bp paired‐end mode. Finally, we obtained 367,645,500 reads for *G. nipponicus* and 362,743,836 reads for *G. aculeatus* .

From the HiFi and Omni‐C reads, two sets of haplotype‐resolved contigs (haplotigs) were generated using *Hifiasm* v0.19.9 with the default parameters of Hi‐C integration (Cheng et al. 2021, 2022). The obtained haplotig sets were further scaffolded by using Omni‐C reads. To this end, Omni‐C reads were trimmed to remove adapter sequences and low‐quality reads by *fastp* v0.23.4 with the following parameters: qualified_quality_phred 30 and unqualified_percent_limit 80 (Chen et al. 2018). Then, filtered Omni‐C reads were mapped to each haplotig set using *bwa‐mem2* v2.2.1 with the parameters recommended for Omni‐C (https://omni‐c.readthedocs.io/en/latest/fastq_to_bam.html) (Li 2013; Md et al. 2019). *Pairtools* was used to record valid Hi‐C read pairs, remove PCR duplicates, and generate bam files (Open2C et al. 2024). *YaHS* v1.2 software was used for scaffolding haplotigs (Zhou et al. 2023). Obtained scaffolds were further manually curated using *Juicebox* v1.11.08 (Dudchenko et al. 2018; Durand, Robinson, et al. 2016).

The directions and names of chromosome‐scale scaffolds followed those of the stickleback version 5 genome (Nath et al. 2021). For each autosome, we selected one chromosome‐scale haplotype with fewer gaps, and chromosome‐scale X and Y chromosomes were also selected for each species. These were used as reference sequences of the two species in this study. These genome assemblies were termed PO v20240517 for *G. aculeatus* and JS v20240517 for *G. nipponicus*. The quality of assembly was evaluated by BUSCO v5.1.2 implemented in *gVolante* v2 using the ortholog set of Actinopterigii in *OrthoDB* v10 (Kriventseva et al. 2019; Manni et al. 2021; Nishimura et al. 2017, 2019).

We also made hardmasked versions of the reference genomes to avoid reduction of mapping quality by multi‐mapping. First, as the pseudo‐autosomal region (PAR) is included in both X and Y chromosomes and is redundant, we hardmasked PAR on the Y chromosome. For each species, X and Y chromosomes were aligned by *minimap2* v2.28, followed by visualisation with dotplot using *D‐Genies* v1.5.0 (Cabanettes and Klopp 2018; Li 2018, 2021). PARs were defined as homologous regions between X and Y with an identity score exceeding 0.5 in *D‐Genies* and without any apparent structural variation located at the first several megabases (Ross and Peichel 2008; White et al. 2015). We defined the first 3,207,000 bp of chrY_IX of *G. nipponicus* and the first 3,236,000 bp of chrY of *G. aculeatus* as PARs, which were masked by Ns. Second, because some regions of the neo‐Y part of the fused Y chromosome (chrY_IX) of *G*. *nipponicus* have not degenerated and share sequences with the neo‐X chromosome (chrIX of *G. nipponicus*) (Yoshida et al. 2014, 2017), the 21,723,000–44,829,387 bp region on chrY_IX was also hardmasked. Third, highly repetitive sequences on chrXII, including 5S ribosomal RNA genes, were hardmasked. Using the tandem repeat‐detecting software *TRASH* v1.2 (Wlodzimierz et al. 2023), we hardmasked the genomic regions of 20,676,000–21,892,999 bp and 22,110,000–29,005,999 bp on chrXII for *G*. *nipponicus* and 21,026,999–23,662,723 bp on chrXII for *G. aculeatus* . Fourth, a highly variable region was found on chrXXI in both species. This region did not show clear alignment even between haplotypes (Figure S3). We defined this region using *D‐Genies* and hardmasked the 580,259–3,826,000 bp region of *G*. *nipponicus* chrXXI and the 4,361,000–8,588,000 bp region of *G. aculeatus* chrXXI. As a result, we obtained masked genome assemblies for *G. aculeatus* (PO v20240517‐masked) and *G. nipponicus* (JS v20240517‐masked).

### 2.2 Genome Annotation

We annotated two haplotype genomes separately for each species. We used the annotation pipeline described previously (Seiko et al. 2024). First, we conducted gene annotation using the *GeMoMa* v1.9 pipeline (Keilwagen et al. 2016, 2018) and a protein database with annotations for southern bluefin tuna (fThuMac1.1), clown anemonefish (ASM2253959v1) (Ryu et al. 2022), Japanese medaka (ASM223467v1) (Ichikawa et al. 2017), three‐spined stickleback (GAculeatus_UGA_version5) (Nath et al. 2021), and Nile tilapia (O_niloticus_UMD_NMBU) (Conte et al. 2017). RNA‐seq data of the two stickleback species (Yoshida et al. 2019) were also used for gene annotation by GeMoMa. RNA‐seq reads were first de novo assembled by *Trinity* v2.15.1 (Grabherr et al. 2011) and then mapped back to the assembled transcriptome by *Hisat2* v2.2.1 (Kim et al. 2015, 2019) to remove any minor transcripts (Seiko et al. 2024). These RNA‐seq reads were mapped to the genome assemblies by *Hisat2* and used as the files for input into *GeMoMa*. Second, we annotated repetitive sequences to use the repeat masked genome for the following gene prediction analysis by *BRAKER2*. *RepeatModeler* v2.0.5 with the *LTRStruct* option was used for the identification and modelling of de novo transposable element families (Flynn et al. 2020). Repeat sequence database of *RepeatModeler*, dfam database v38 (Storer et al. 2021), and Repbase database (RepBaseRepeatMaskerEdition‐20181026) (Bao et al. 2015) were used for soft masking by *RepeatMasker* v4.1.6 (https://www.repeatmasker.org/RepeatMasker/). To find any other genes that were not previously annotated, we applied the gene prediction pipeline *BRAKER2* with the *OrthoDB* v11 Vertebrata protein sequence set to the soft‐masked genome assembly (Brůna et al. 2021; Kuznetsov et al. 2023). Genes newly detected in *BRAKER2* were also added.

For further validation of the annotated protein sequences, we used *EnTAP* v1.0.1 (Hart et al. 2020), which searches for homologous proteins using a database. For this, we used RefSeq vertebrate_other, uniprot_sprot, and uniprot_tremble as databases (The UniProt Consortium 2023; O'Leary et al. 2016). Genes with hits in “Fishes,” “Aves,” “Mammals,” “Animals,” or “Eukaryotes” in the EggNOG Tax Scope column were considered to be real gene products and selected. Common gene names were added by *EggNOG mapper* v2.1.12 (Cantalapiedra et al. 2021). We evaluated the completeness of the peptide sequences of the predicted gene set with BUSCO implemented in *gVolante*.

### 2.3 Testing the Association of Chromosomal Inversions With Levels of Genetic Differentiation and Introgression

To detect chromosomal inversions, we first conducted pairwise alignment between the *G. nipponicus* assembly (JS v20240517) and the Japanese *G. aculeatus* assembly (PO v20240517). For alignment, *minimap2* v2.28‐r1209 (Li 2018, 2021) was used to map the *G. nipponicus* assembly as a query and the *G. aculeatus* assembly as a reference with the *‐x asm5* option. *Syri* v1.7.0 was used to detect structural variations for each pairwise alignment (Goel et al. 2019). The output of *syri* was visualised by *plotsr* v1.1.1 (Goel and Schneeberger 2022). We extracted inversion regions and colinear regions from the *syri* output vcf file. For inversions, variants with ID assigned as INV + number were extracted. Meanwhile, for colinear regions, variants with ID assigned as SYNAL + number were extracted. We excluded Y and neo‐Y chromosomes from the analysis of inversions.

To investigate whether genomic differentiation and divergence between the sympatric Japanese species are greater in the inverted regions than in the colinear ones, we used previously generated short‐read sequences of a pair of these two species living in sympatry in the Bekanbeushi River system, Hokkaido, Japan (10 individuals for each species) (Yoshida et al. 2014) (Table S1). Additionally, to investigate introgression patterns, we conducted the ABBA‐BABA test (see below). For this test, we used the publicly available short reads of 10 three‐spined stickleback individuals in Quebec, Canada, as an allopatric population (Sylvestre et al. 2023), and one individual of *G. wheatlandi* as an outgroup (Yoshida et al. 2014) (Table S1).

All short reads were cleaned by *fastp* v0.23.2 with the following settings: ‐‐*detect_adapter_for_pe*, *‐‐cut_right*, *‐‐cut_window_size* 4, *‐‐cut_mean_quality* 20, *‐l* 35. Then, we mapped the cleaned fastq files to the masked Japanese three‐spined stickleback genome assembly (PO v20240517‐masked) using *bwa‐mem2* v2.2.1 with the default settings. SNPs were called by the *bcftools* v1.9 *mpileup* and *call* pipeline (Danecek et al. 2021; Li 2011).

To calculate *F*_ST_ and *d*_xy_ within inversions and colinear regions separately, we filtered out low‐quality sites using *vcftools* with the following options: *‐‐max‐meanDP* 45.09087 (mean depth + 4 × √mean depth) (Li 2014), *‐‐remove‐indels*, *‐‐max‐alleles* 2, *‐‐minQ* 30, *‐‐minDP* 10, *‐‐minGQ* 30, *‐‐max‐missing* 0.8 (Danecek et al. 2011). We also classified sites within inversions and colinear regions using vcftools. We calculated the statistics for each 25 kbp window. When a window contained both inverted and colinear regions, we calculated *F*_ST_ and *d*_xy_ separately for the inverted and colinear regions. *Pixy* was used to calculate Weir and Cockerham's *F*_ST_ and *d*_xy_ (Korunes and Samuk 2021). We only used windows with 25 or more genotyped SNPs for *F*_ST_, and windows with 2500 or more sites for *d*_xy_. Sex chromosomes (chrIX, chrXIX, and chrY) were excluded from the calculation.

To compare introgression patterns between inverted and colinear regions, we conducted the ABBA‐BABA test. We first extracted sites with no missing genotype in *G. wheatlandi* using *vcftools* with the following settings: *‐‐max‐meanDP* 45.09087, ‑‑*remove‐indels*, *‐‐minQ* 30, *‐‐minDP* 10, *‐‐minGQ* 30, *‐‐max‐missing* 1. Next, at these selected sites, we applied filtering to remove low‐quality sites by *vcftools* with the following options: *‐‐max‐meanDP* 45.09087, *‐‐remove‐indels*, *‐‐min‐allels* 2, ‑‑*max‐alleles* 2, *‐‐mac* 1, *‐‐positions G. wheatlandi* site positions, *‐‐minQ* 30, ‑‑*minDP* 10, *‐‐minGQ* 30, *‐‐max‐missing* 0.8. Sex chromosomes (chrIX, chrXIX, and chrY) were also removed. At the same time, we separated SNPs within inversions and colinear regions via the above filtering step. *Dsuite* was used to calculate *D*‐statistics (Malinsky et al. 2021). We set *G. aculeatus* as P2, *G. nipponicus* as P3, and the allopatric *G. aculeatus* in Quebec as P1. Whether *D* was significantly different from zero was tested by a standard block‐jackknife procedure.

### 2.4 3D Genome Analysis

For 3D genome analysis, we separately obtained high‐coverage Hi‐C data with DpnII and HinfI restriction enzymes based on the iconHi‐C protocol (Kadota et al. 2020). We used different female individuals of the same populations of the two species (one fish per species). Muscle tissue weighing 126 mg for *G. nipponicus* and 133 mg for *G. aculeatus* was powderized in liquid nitrogen with a mortar and pestle, and subsequently used as the input material for Hi‐C. Illumina‐compatible libraries were generated using the KAPA LTP Library Preparation Kit (Roche, Switzerland) with IDT for Illumina‐TruSeq DNA UD Indexes (Illumina). The prepared Hi‐C libraries were sequenced on the HiSeq X Ten platform (Illumina) in Novogene (~1 lane for each species), and 449,599,747 or 449,628,420 million paired‐end 150 bp read pairs were obtained for *G. nipponicus* and *G. aculeatus* , respectively.

We first obtained chromatin contact matrices for each species using the same pipeline recommended for Omni‐C (https://omni‐c.readthedocs.io/en/latest/fastq_to_bam.html). The reads were mapped to the masked reference genomes prepared above (JS v20240517‐masked and PO v20240517‐masked). Library quality control was conducted by *pairtools*. Obtained .*pair* format files were converted to .*hic* format files by *juicertools* v1.22.01 (Durand, Shamim, et al. 2016), and further converted to .*mcool* format files by the *hicConvertFormat* command implemented in *HiCexplorer* v3.7.3 (Ramírez et al. 2018).

We detected A/B compartments by *FAN‐C* v0.9.1 (Kruse et al. 2020). First, Knight‐Ruiz (KR) normalisation was applied to the contact matrices using the *fanc hic* command. Next, the *fanc compartments* command was used to calculate principal components (PCs) based on Pearson's correlation matrix. Bin size was set to 100,000 bp. *FAN‐C* automatically assigns positive eigenvector scores to the regions with higher GC contents and infers that those regions are A compartments, whereas other regions with negative scores are inferred to be B compartments. The obtained Pearson's correlation matrix and eigenvectors of PC1 were plotted using the *fanc plot* command.

To compare gene expression levels between A and B compartments, we first classified genes into those in A compartments and those in B compartments. Genes overlapping with both A and B compartments, namely, spanning their boundaries, were removed from the analysis. Yoshida et al. (2019) reported RNA‐seq reads of muscle tissues from the two stickleback species (*N* = 7 for each species). We first filtered low‐quality reads and adapter sequences by *fastp* with the default settings, and then mapped them to the masked genome of each species using *STAR* v.2.7.11 (Dobin et al. 2013). *Featurecount* v.2.0.6 was used to extract mapped read counts (Liao et al. 2014). Read counts were then normalised by the transcripts per million (TPM) method with a custom *R* script. We took the average of TPM among individuals for each gene and compared the average TPM between A and B compartments using the Brunner–Munzel test.

To detect TADs, we used *SpectralTAD* software (Cresswell et al. 2020) because previous benchmark research demonstrated that it provides reproducible results regardless of the data volume and bin size (Xu et al. 2024). We used a non‐normalised count matrix as recommended by the manual of *SpectralTAD* (Cresswell et al. 2020) and set the bin size as 25,000 bp. The results were viewed using *HiGlass* v.1.13.4 (Kerpedjiev et al. 2018). The TAD hierarchy level to be estimated was set to 2. We followed the definition of TAD domains proposed in previous studies (An et al. 2019; Cresswell et al. 2020). Briefly, TADs not nested within other TADs were defined as primary TADs, whereas TADs nested within other TADs were defined as secondary TADs. *SpectralTAD* sometimes called the same TAD both a primary TAD and a secondary TAD. In such cases, we treated it as a primary TAD. Although An et al. (2019) and Cresswell et al. (2020) defined TAD boundaries as the rightmost point of a given TAD, we here considered both leftmost and rightmost points of either primary or secondary TADs as TAD boundaries.

### 2.5 Analysis of the Relationship Between TADs and Inversions

We first investigated whether TAD boundaries are more associated with inversion breakpoints than other regions. Inversions detected as described in Section 2.3 were used. We prepared two inversion datasets for the *G. nipponicus* and *G. aculeatus* pair. The first one included only inversions longer than the bin size used for the TAD analysis (25 kbp), whereas the other included inversions of all sizes. We then calculated the distance between each inversion breakpoint and the nearest TAD boundary.

We also conducted the same analysis for the inversions between our genome assembly and the publicly available North American freshwater stickleback reference genome (version 5; Nath et al. 2021). Inversions were detected by the same method as described in Section 2.3. In this analysis, we focused on the previously reported three inversions on chrI, chrXI, and chrXXI because they may be associated with genetic differentiation between freshwater and marine populations (Jones et al. 2012). We also similarly analysed the distance between inversion breakpoints and boundaries of A/B compartments.

## 3 Results

### 3.1 Chromosomal Genome Assemblies of *G. nipponicus* and Japanese *G. aculeatus*

**FIGURE 1:**
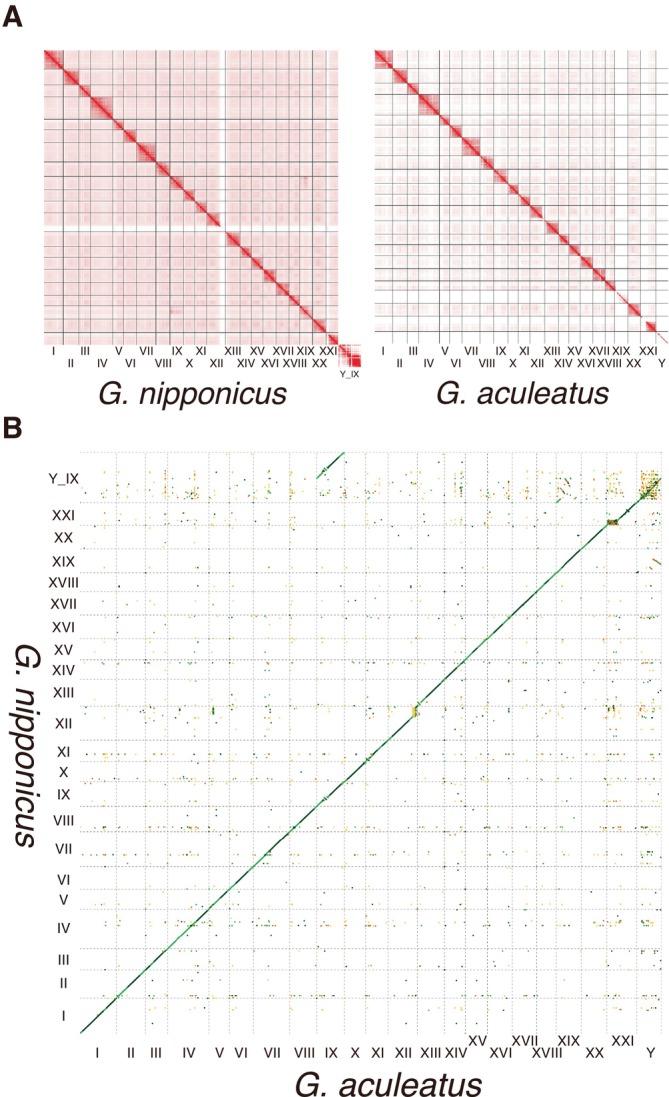
Genome assemblies of *Gasterosteus nipponicus* and *Gasterosteus aculeatus* . Omni‐C contact map of *G. nipponicus* and *G. aculeatus* (A). (B) Dotplot between *G. nipponicus* and *G. aculeatus* .

We successfully constructed chromosome‐scale haplotype‐phased assemblies of both species (Table 1; Figure 1A, Figures S1 and S2). The level of contiguity evaluated by the number of scaffolds for each assembled haplotype set was very high in both species (Table S2): 100 and 69 for each haplotype set of *G. nipponicus* and 117 and 160 for that of *G. aculeatus* . Two haplotypes of the same chromosome were highly colinear within an individual, indicating the robustness of the haplotype phasing (Figure S3). Upon selecting one of the two haplotypes with fewer gaps for each autosome, we obtained 22 chromosome‐scale scaffolds, including 20 autosomes, the X chromosome, and the Y chromosome. There were only 93 gaps for *G. nipponicus* (JS v20240517), but 56 gaps for *G. aculeatus* (PO v20240517) (Table 1). Focusing on Y chromosomes, which are difficult to assemble in general because of the high repeat contents (Peichel et al. 2020), there were only 9 gaps for the *G. nipponicus* neo‐Y chromosome and one gap for the *G. aculeatus* Y chromosome. The percentages of single‐copy orthologs detected as complete in the genome assemblies by BUSCO were 98.1% for *G. nipponicus* and 98.1% for *G. aculeatus* , whereas those for predicted protein‐coding genes were 99.04% for *G. nipponicus* and 99.01% for *G. aculeatus* , indicating a high coverage of the protein‐coding landscape (Table 1).

**TABLE 1:** Statistics of genome assembly and gene annotation.

	*Gasterosteus nipponicus*	*Gasterosteus aculeatus*
Genome assembly		
Assembly size (bp)	519,334,188	501,984,537
Number of scaffolds	23	23
N50 length (bp)	22,062,446	22,172,610
Number of gaps	93	56
BUSCO complete (%)	98.1	98.1
BUSCO complete + fragmented (%)	98.63	98.71
Annotated protein‐coding genes		
Number of genes	27,059	26,646
BUSCO complete (%)	99.04	99.01
BUSCO complete + fragmented (%)	99.31	99.45

### 3.2 Inversions Act as Barriers to Gene Flow

**FIGURE 2:**
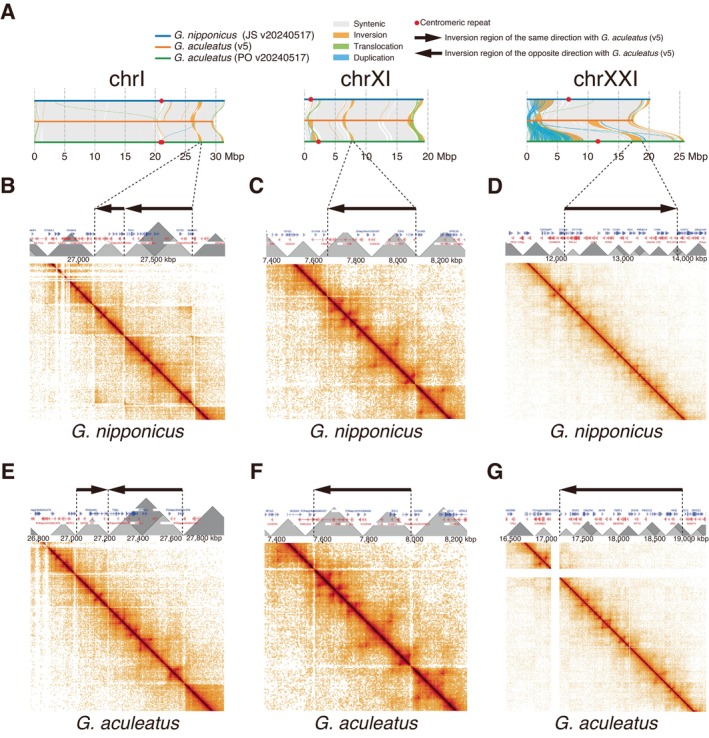
Relationships between inversion breakpoints and TAD boundaries analysed for inversions located on chromosomes I, XI, and XXI. (A) Alignment of chromosomes I, XI, and XXI among genomes assembled in this study (PO v20240517 for Japanese marine *Gasterosteus aculeatus* and JS v20240517 for *Gasterosteus nipponicus*) and the stickleback reference genome (version 5). (B–D) Hi‐C contact maps (below) with TAD structures (above) around the inversions in *G. nipponicus*. (E–G) Hi‐C contact maps (below) and TAD structures (above) around the inversions in Japanese *G. aculeatus* . Arrows indicate the direction of inversions when the *G. aculeatus* v5 reference genome is directed rightward. Dark grey TADs indicate primary TADs, whereas light grey ones indicate secondary TADs.

Genome alignment of the two species showed that most genomic regions were colinear overall (Figure 1B), and the fused sex chromosome of *G. nipponicus* (chrY_IX) also aligned well with the separated Y and chrIX of *G. aculeatus* . Although they were linear overall, we detected 59 inversions between *G. nipponicus* and *G. aculeatus* (Figure S5). A previous study reported three inversions on chrI, chrXI, and chrXXI, which may be associated with genetic differentiation between marine and freshwater stickleback populations (Jones et al. 2012). As expected from the fact that the stickleback reference genome (version 5) is based on freshwater *G. aculeatus* populations and our *G. aculeatus* assembly is based on a Japanese anadromous (sea‐run migratory) fish, all three of these regions showed inversions between the version 5 reference genome and our *G. aculeatus* assembly (Figure 2A). *G. nipponicus*, which is also anadromous, showed inversions on chrI and chrXI relative to the version 5 genome, whereas they were colinear on the chrXXI region, suggesting that this orientation may be ancestral in *Gasterosteus*.

**FIGURE 3:**
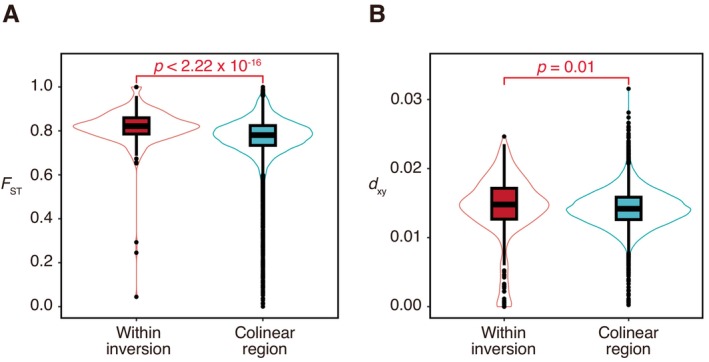
Higher genetic differentiation and divergence between *Gasterosteus nipponicus* and *Gasterosteus aculeatus* within inversions than within colinear regions. (A) Comparison of *F*_ST_ between inverted and colinear regions. (B) Comparison of *d*_xy_ between inverted and colinear regions.

We next investigated whether the inversions act as barriers to gene flow between the sympatric Japanese stickleback species. *F*_ST_ values within the inversions were significantly higher than those within the collinear regions (mean and median *F*_ST_ within inversions = 0.817 and 0.822, and within colinear regions = 0.771 and 0.781, respectively; Brunner–Munzel test, *p* < 2.22 × 10^−16^; Figure 3). A significant difference was also found in *d*_xy_ between the inverted and collinear regions (mean and median *d*_xy_ within inversions = 0.0141 and 0.0148, and within colinear regions = 0.0142 and 0.0142, respectively; Brunner–Munzel test, *p* = 0.01; Figure 3). The analysis using *D*‐statistics did not detect significant gene flow within the inversions (*D* = 2.55 × 10^−2^, *Z*‐score = 0.881, *p* = 0.378), whereas signatures of gene flow were found in the collinear regions (*D* = 0.143, *Z*‐score = 28.3, *p* = 2.3 × 10^−16^). These results indicate that the chromosomal inversions likely act as barriers to gene flow.

### 3.3 Stickleback 3D Genome Structures

**FIGURE 4:**
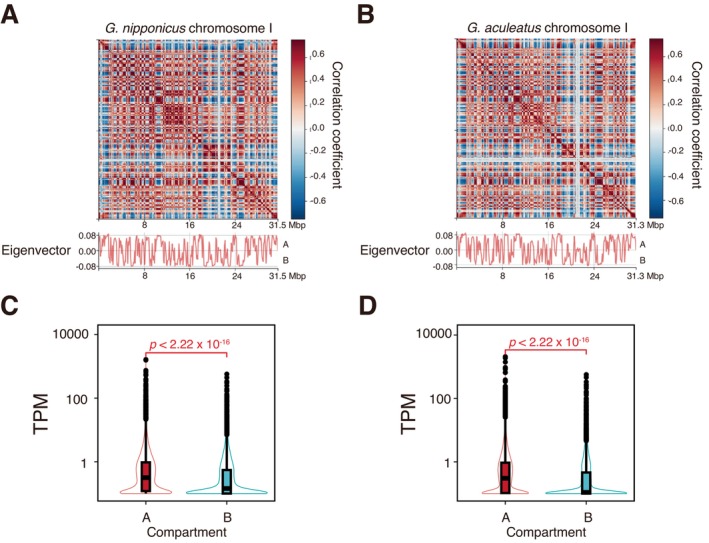
Stickleback A/B compartments. Heatmaps of Pearson's correlation matrix of chromosome I of *Gasterosteus nipponicus* (A) and *Gasterosteus aculeatus* (B) are shown as representative examples. Positive eigenvector values indicate A compartments, whereas negative ones indicate B compartments. (C, D) Gene expression levels measured as transcripts per million (TPM) were compared between A and B compartments in *G. nipponicus* (C) and *G. aculeatus* (D).

Using the high‐coverage iconHi‐C data, we first identified A and B compartments for each species. The proportions of the genome classified into A and B compartments were similar between the species (Table 2). Significantly more genes were included within the A compartment than within the B compartment (*χ* ^2^ test, *χ* ^2^ = 322.8, *p* < 2.2 × 10^−16^ for *G. nipponicus*; and *χ* ^2^ = 531.1, *p* < 2.2 × 10^−16^ for *G. aculeatus* ). Furthermore, genes in the A compartment were more highly expressed than those in the B compartment in both species (Brunner–Munzel test, *p* = 2.22 × 10^−16^ for both species; Figure 4 and Figure S4). These results suggest that the A compartment is an active part of the genome, whereas the B compartment is an inactive one.

**TABLE 2:** Statistics of A/B compartments and TADs in *Gasterosteus nipponicus* and *Gasterosteus aculeatus* .

	*G. nipponicus*	*G. aculeatus*
A/B compartment		
Proportion of A compartment (%)	52.49%	53.70%
Proportion of B compartment (%)	46.47%	44.74%
Average one domain size of A compartment (bp)	478,005.90	470,365.2
Average one domain size of B compartment (bp)	419,201.50	382,238.0
Number of genes assigned to A compartment	13,261	15,199
Number of genes assigned to B compartment	8255	8169
TAD		
Primary TAD count	1358	1357
Primary TAD average size (bp)	328,829.2	330,876.9
Primary TAD media size (bp)	300,000	300,000
Secondary TAD count	717	777
Secondary TAD average size (bp)	230,435.7	228,103.2
Secondary TAD median size (bp)	200,000	200,000
Level 1 TAD boundary count	1459	1472
Level 2 TAD boundary count	308	331

We next investigated TADs and identified 1358 and 1357 primary TADs for *G. nipponicus* and *G. aculeatus* , respectively (Table 2). These counts were similar to the previously reported TAD counts in zebrafish, namely, 1238–1350 with 10 kbp bin size (Yang et al. 2020). The median sizes of primary TADs were 328.8 and 330.9 kbp for *G. nipponicus* and *G. aculeatus* , respectively, which were slightly smaller than that of zebrafish, but within the same order of magnitude (~500 kbp with a 20 kbp bin size) (Kaaij et al. 2018).

### 3.4 Inversion Breakpoints Overlap With TAD Boundaries

To investigate the relationship between the inversion breakpoints and TAD boundaries, we first examined the three previously reported inversions on chrI, chrXI and chrXXI (Jones et al. 2012). All breakpoints of these three inversions except the left boundary of the chrXI region were located within 25 kbp (1 bin size) from the nearest TAD boundary (Figure 2B‐G). Even for the one exception on chrXI, the distance between the inversion breakpoint and a nearby TAD boundary was 41.75 kbp (1.67 bin size) in *G. nipponicus* and 37 kbp (1.48 bin size) in *G. aculeatus* .

**FIGURE 5:**
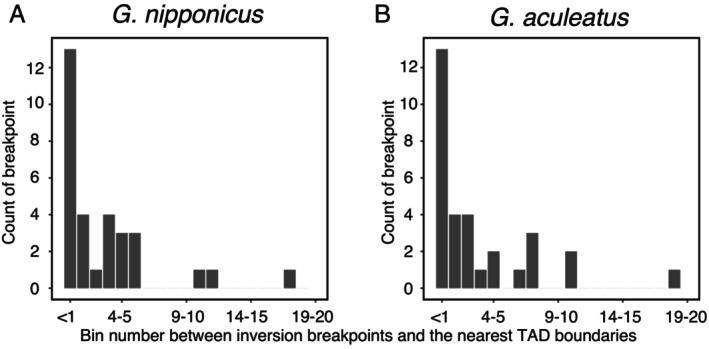
Distance between inversion breakpoints and TAD boundaries. The coordinates of TADs are based on *Gasterosteus nipponicus* (A) and *Gasterosteus aculeatus* (B). Sixteen inversions larger than 25 kbp were used (32 breakpoints in total). Distance was converted to bin size (1 bin = 25 kbp).

We next examined the inversions detected between our *G. aculeatus* and *G. nipponicus* assemblies. Focusing on 16 large inversions larger than 25 kbp, at least one side of the inversion breakpoints overlapped with a TAD boundary in 10 of these 16 large inversions for both species. In three inversions, both ends of the breakpoints overlapped with TAD boundaries. If inversion breakpoints were randomly distributed in the genome, the expected frequency of breakpoints located within 1 bin of the nearest TAD boundary would be 20.0% for *G. nipponicus* and 20.5% for *G. aculeatus* . However, the observed frequency was 40.6% (13/32) for both species, which was much higher than expected (Figure 5A, Table S3). *χ* ^2^ test confirmed that this overlap was significant for both species (*χ* ^2^ = 12.16, *p* = 4.883 × 10^−4^ for *G. nipponicus*; and *χ* ^2^ = 16.402, *p* = 5.124 × 10^−5^ for *G. aculeatus* ). Using all 59 inversions, the observed frequency of overlap between inversion breakpoints and TAD boundaries was 28.0% in *G. nipponicus* (*χ* ^2^ = 10.122, *p* = 1.465 × 10^−3^) and 33.9% in *G. aculeatus* (*χ* ^2^ = 20.605, *p* = 5.644 × 10^−6^; Figure S6). These data suggest that inversion breakpoints tend to occur near TAD boundaries.

We also analysed the association between inversion breakpoints and A/B compartment boundaries. The expected and observed frequencies of breakpoints of large inversions (> 25 kbp) located within 25 kbp from the nearest A/B compartment boundary were 11.69% and 20.59% in *G. nipponicus* (*χ* ^2^ = 5.5117, *p* = 0.01889), and 12.79% and 21.88% in *G. aculeatus* (*χ* ^2^ = 3.9865, *p* = 0.04587), respectively (Figure S7A,B), suggesting that large inversions tend to occur in association with A/B compartment boundaries. Analysing all inversions, the observed frequencies—14.4% in *G. nipponicus* (*χ* ^2^ = 1.5959, *p* = 0.2065) and 11.86% in *G. aculeatus* (*χ* ^2^ = 0.073858, *p* = 0.7858)—were similar to or lower than the expected frequencies (Figure S7C,D).

## 4 Discussion

### 4.1 Newly Assembled Genomes of Two Japanese *Gasterosteus* Sticklebacks

We successfully obtained haplotype‐phased chromosome‐scale genome assemblies for *G. nipponicus* and *G. aculeatus* . Key assembly statistics, including the number of gaps, contig N50 length, and assembly size, showed significant improvements compared with those of the previous reference genomes of *G. aculeatus* (version 5) (number of gaps = 3144, contig N50 = 510.82 kbp) (Nath et al. 2021) and *G. nipponicus* (number of contigs = 3095, contig N50 = 565 kbp, scaffold N50 = 12.71 Mbp) (Yoshitake et al. 2022) (Table S2). These improvements were achieved through the use of advanced haplotype‐phased assembly methods, employing PacBio HiFi sequencing and Dovetail Omni‐C technologies. Our assemblies revealed two highly repetitive regions on chrXII and chrXXI, which have not been identified previously. The high contiguity and completeness of these genome assemblies ensure the accuracy of the inversions detected between the two species. Although the present study focused on the analysis of chromosomal inversions on autosomes, these assemblies are useful for further analysis on other types of mutations, such as copy number variations and indels, which are important for adaptation in sticklebacks (Ishikawa et al. 2019; Xie et al. 2019), and on sex chromosome evolution, which is involved in reproductive isolation between these two species (Kitano et al. 2009).

### 4.2 Inversions May Act as Barriers to Gene Flow Between Sympatric Species

Our *D*‐statistic analysis did not detect gene flow within inversions, despite its occurrence in colinear regions. The *F*_ST_ value was significantly higher within inversions. The *d*_xy_ value was also significantly, but slightly higher within inversion, though we cannot exclude the possibility that this significance is due to a large number of data points. Although these regions did not exhibit the localised divergence typically referred to as ‘genomic islands of speciation’ (Ravinet et al. 2018), this is likely because overall genetic differentiation between these two species is high. This high level of differentiation is probably due to them having diverged a long time ago and the low rate of gene flow between them for most of the genome (Ravinet et al. 2018). Thus, these results likely indicate the significant reduction of either current, past or both gene flow within inversions, and likely support the well‐supported concept that inversions act as barriers to gene flow between sympatric species.

Currently, we do not know whether genes within the inversions indeed contribute to local adaptation or reproductive isolation. Although a previous study showed that alleles within the three inversions on chrI, chrXI and chrXXI are genetically differentiated between marine and freshwater populations within *G. aculeatus* (Jones et al. 2012), they do not substantially overlap with quantitative trait loci (QTLs) responsible for morphological differences between marine and freshwater populations (Miller et al. 2014; Peichel and Marques 2017). In the pair of Japanese stickleback species investigated in this study, several QTLs for reproductive isolation are localised on sex chromosomes (Kitano et al. 2009), but we do not yet know whether QTLs for any other morphological and physiological differences overlap with the autosomal inversions identified in this study. Because these two species differ in many traits and multiple isolating barriers act to prevent gene flow between them (Kitano et al. 2007, 2009), further genetic studies on them should help to resolve this issue.

Here, we found that the chrXXI region of *G. nipponicus* was colinear with that of freshwater populations of *G. aculeatus* and reversed compared with that of marine populations of *G. aculeatus* , suggesting that this orientation may be ancestral. By performing comparisons with other outgroup species, such as *Pungitius* sticklebacks, we will be able to test this possibility.

### 4.3 Inversion Breakpoints Tend to Occur at TAD Boundaries

Here, we revealed 3D genome structures of *G. nipponicus* and *G. aculeatus* . We found both A/B compartments and TAD structures like in other vertebrates, including a few Actinopterygii species (Hoencamp et al. 2021; Yang et al. 2020). Our study revealed that the breakpoints of inversions between *G. nipponicus* and *G. aculeatus* frequently overlapped with TAD boundaries (Figure 5, Table S3). It remains elusive whether this is due to purifying selection against inversion breakpoints within TADs (Krefting et al. 2018; Lazar et al. 2018; Liao et al. 2021) or the mutagenic nature of the TAD boundaries (Berthelot et al. 2015). It also remains unknown whether this overlap can also be observed in germline cells. Although TAD structures are often conserved across tissues (Dixon et al. 2012, 2016), they may be depleted during spermatogenesis (Zheng and Xie 2019) but see (Vara et al. 2021). If TAD boundaries are mutagenic, other mutations, such as nucleotide substitutions and indels, may also be common there (Guo et al. 2018).

Our study revealed that the breakpoints of inversions, which act as barriers to gene flow between *G. nipponicus* and *G. aculeatus* , tend to occur at TAD boundaries. This trend was particularly pronounced for larger inversions (> 25 kbp in this study) (Figure 5, Figure S7). These findings suggest that TAD structures likely play a role in shaping the genomic landscape of divergence between closely related species. Furthermore, previous studies have shown that inversions can change TAD structures, which can influence gene expression changes and morphological evolution (Galupa et al. 2022; Marlétaz et al. 2023). Our data should be useful for testing whether chromosomal inversions can change TADs and the expression of nearby genes, leading to ecological divergence and speciation in closely related species.

## 5 Conclusions

In the present study, using two Japanese stickleback species, *G. nipponicus* and *G. aculeatus* , we found that inversions likely act as barriers to gene flow and that TAD boundaries tend to overlap with inversion breakpoints. Thus, the 3D genome structure indirectly influences the genomic landscape of divergence possibly by biasing the rates of inversion mutations involving TAD boundaries. Further integration of 3D genome analyses with population genomics has the potential to provide novel insights into the mechanisms by which the 3D genome influences speciation processes.

## Author Contributions

Y.Y.Y. and J.K. designed the study. A.T. conducted HiFi and Omni‐C data acquisition for genome assembly. M.K. and S.K. conducted Hi‐C data acquisition for 3D genome analysis. Y.Y.Y. conducted the bioinformatic analysis. Y.Y.Y. and J.K. wrote the first draft of the manuscript. All authors reviewed the manuscript.

## Conflicts of Interest

The authors declare no conflicts of interest.

## Supporting information


Figure S1‐S7

Table S1‐S4

## Data Availability

All raw sequence data are available from DDBJ: *G. nipponicus* HiFi and Omni‐C data, PRJDB19945; *G. aculeatus* HiFi and Omni‐C data, PRJDB19949; and iconHi‐C for *G. nipponicus* and *G. aculeatus* , PRJDB19958. The following data are available from Dryad (https://doi.org/10.5061/dryad.ncjsxkt65): genome assemblies (JSv20240517, POv20240517, and their masked versions) and their annotations (genes, centromere positions, gap positions, inversions, A/B compartments, TADs), and assemblies and annotations of each haplotype. Codes are available at our GitHub repository (https://github.com/yoyyamasaki/3D_genome_inversions_and_gene_flow_barrier).
