# Supplementary material for: 3D Genome Constrains Breakpoints of Inversions That Can Act as Barriers to Gene Flow in the Stickleback
Source: Mol Ecol. 2025 May 31;34(21):e17814. doi: 10.1111/mec.17814 (PMC12573730; doi:10.1111/mec.17814)
Supplement: Supplementary file 1 — Figure S1‐S7 [file MEC-34-e17814-s001.docx]

**Supplemental Information for:**

**3D Genome Constrains Breakpoints of Inversions that Can Act as Barriers to Gene Flow in the Stickleback**

Yo Y. Yamasaki, Atsushi Toyoda, Mitsutaka Kadota, Shigehiro Kuraku, Jun Kitano

**Table of Contents:**

| **Figure S1** | Page 2 |
| --- | --- |
| **Figure S2** | Page 3 |
| **Figure S3** | Page 4 |
| **Figure S4** | Page 5 |
| **Figure S5** | Page 6 |
| **Figure S6** | Page 7 |
| **Figure S7** | Page 8 |


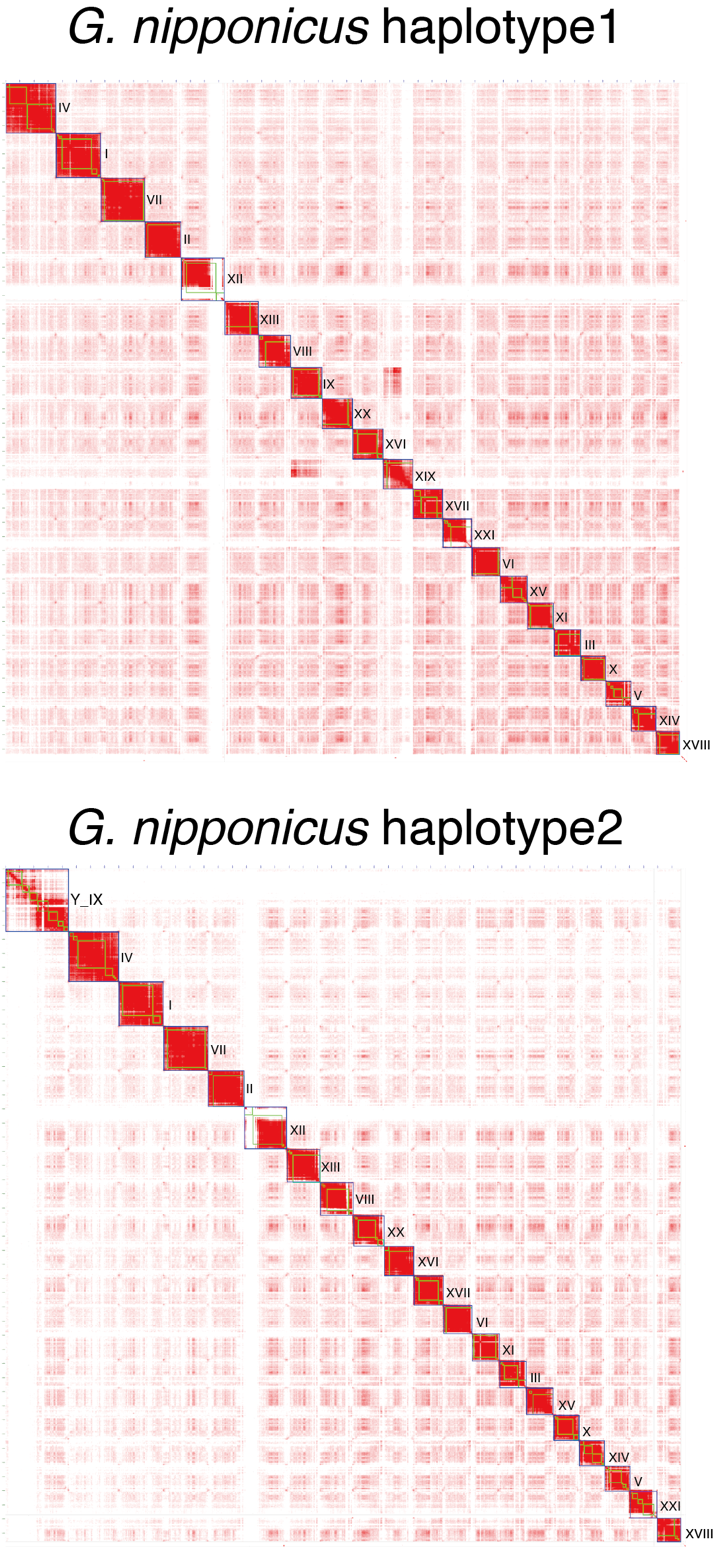


Figure S1

Contact maps of Omni-C reads for each haplotype of *G. nipponicus*.


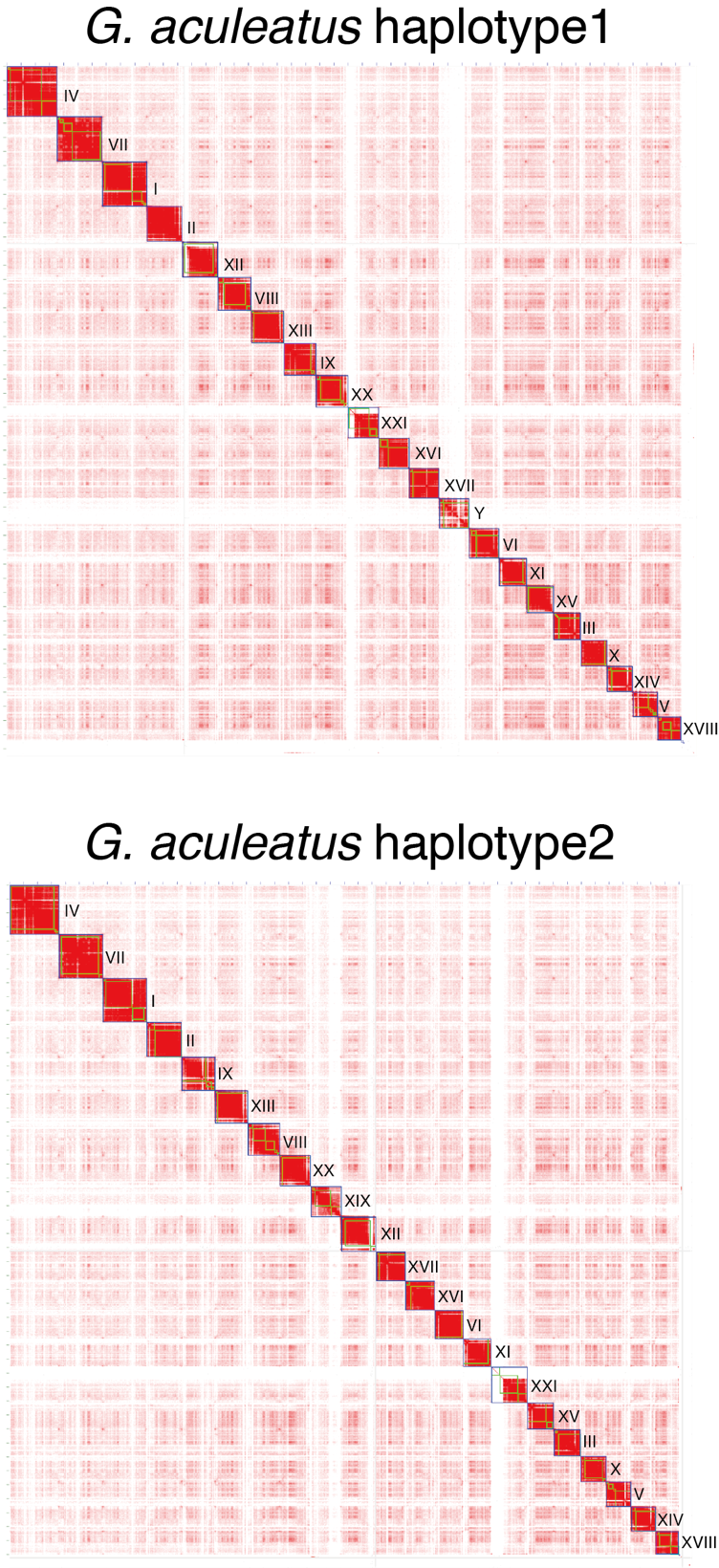


Figure S2

Contact maps of Omni-C reads for each haplotype of *G. aculeatus*.


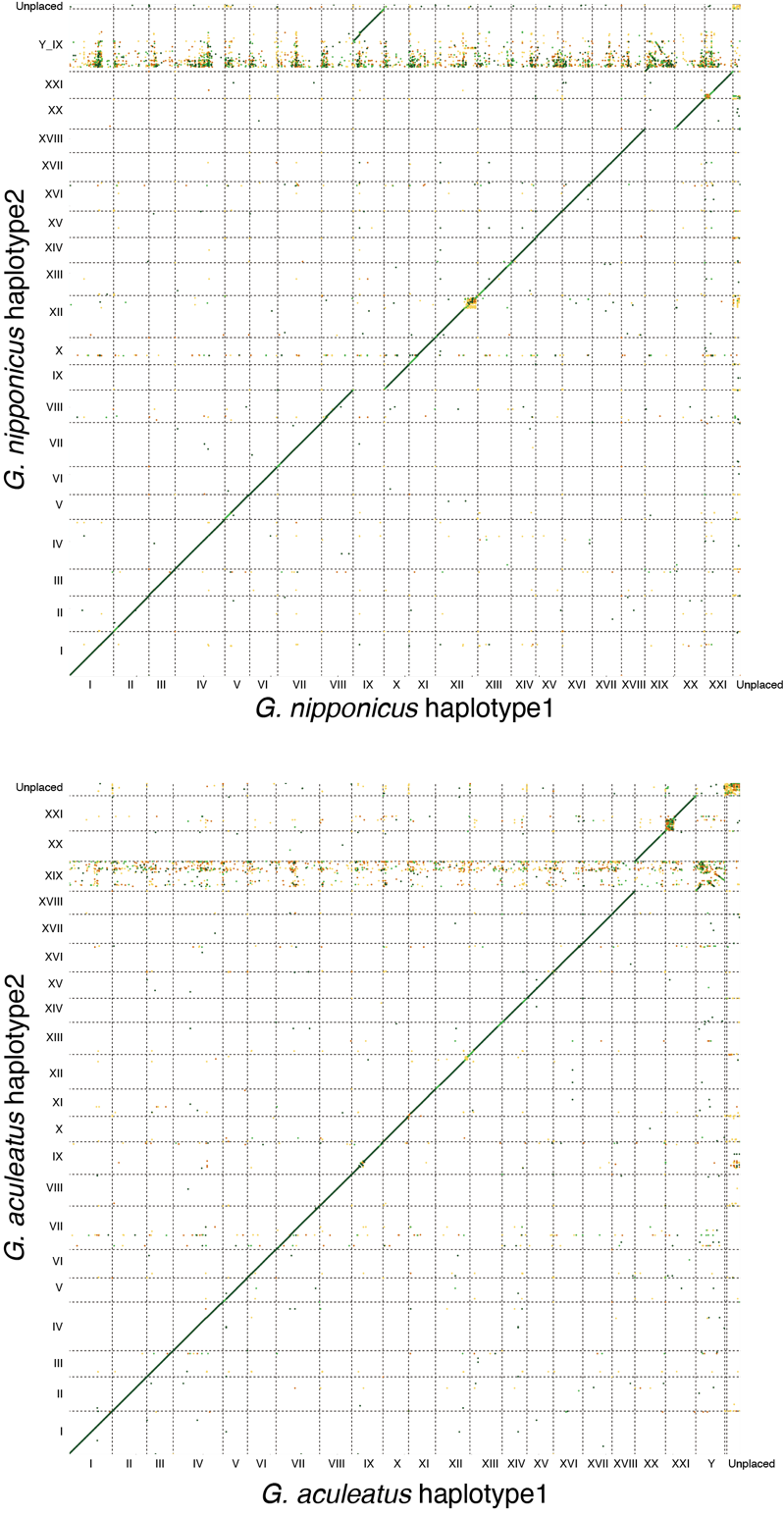


Figure S3

Dotplots between haplotypes within individuals.


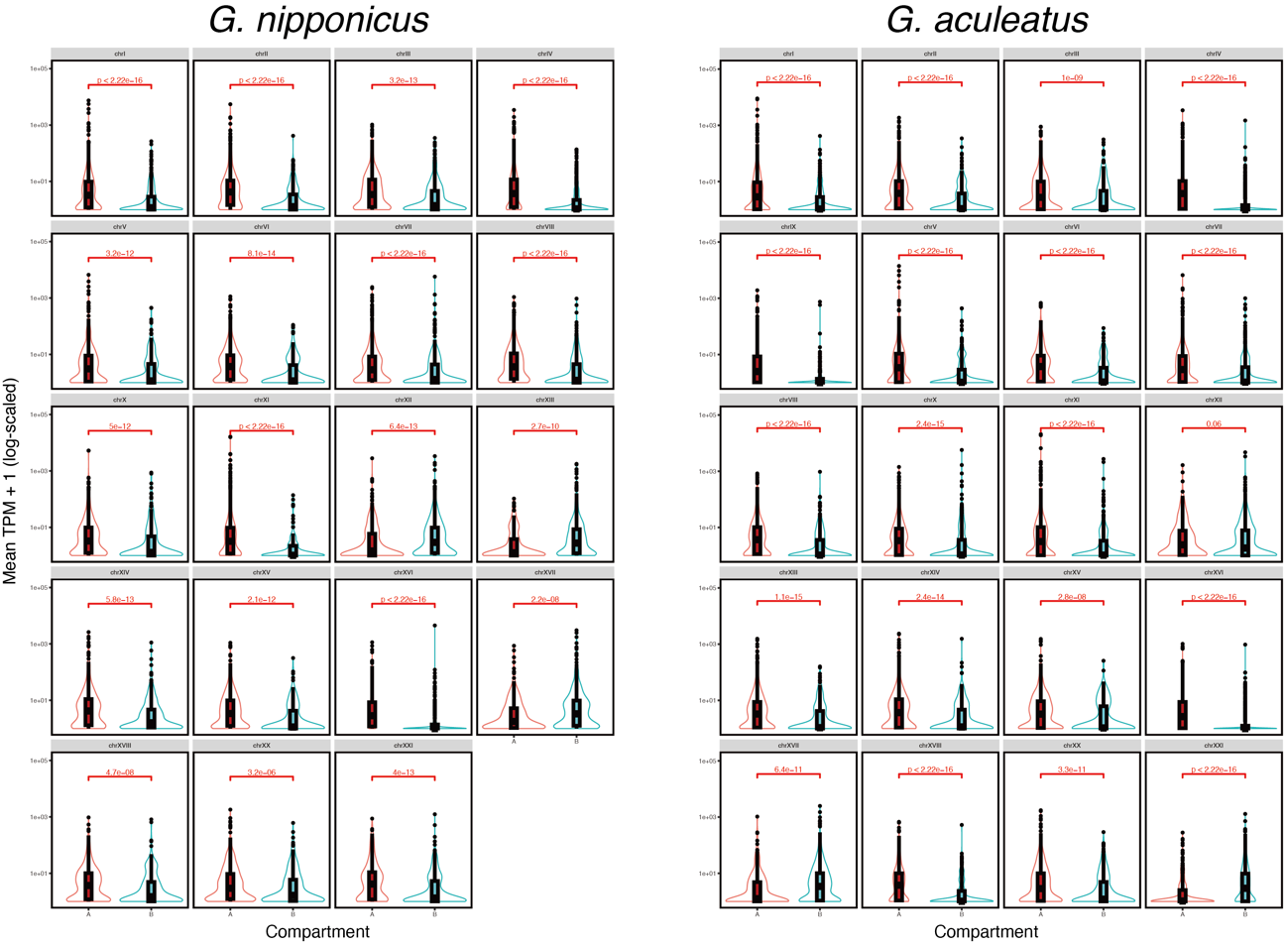


Figure S4

Comparison of gene expression levels between A and B compartments for each chromosome. A/B compartments of *G. nipponicus* (left) and *G. aculeatus* (right) were used here. Higher expression levels for B compartments were observed in chrXIII and chrXVII of *G. nipponicus* and chrXVII and chrXXI of *G. aculeatus*. This may have been due to misclassification of the compartments by the *fanc* program.


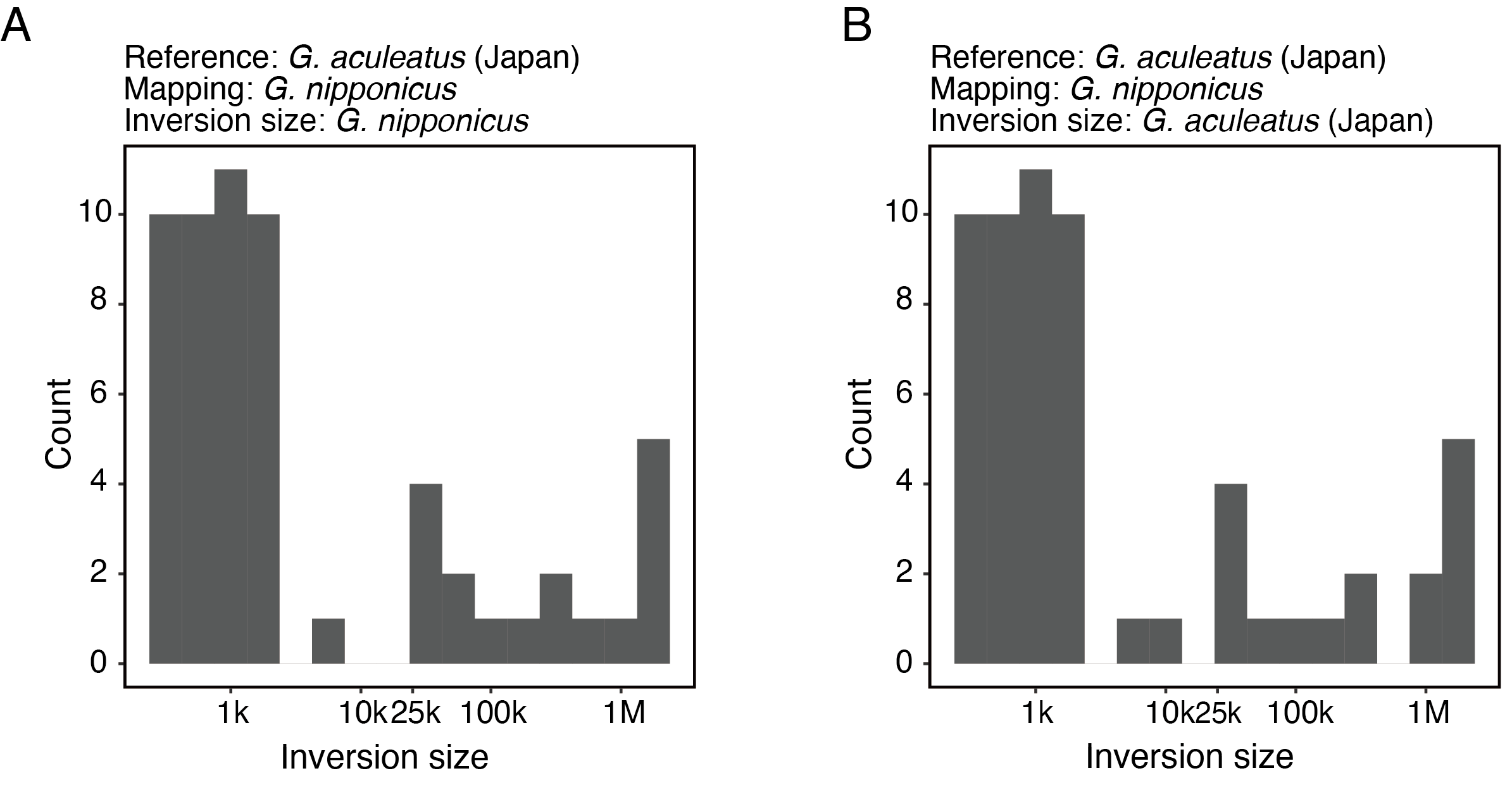


Figure S5

Size distribution of inversions between *G. nipponicus* and *G. aculeatus*. Sizes were calculated on the basis of *G. nipponicus* assembly (A) or *G. aculeatus* assembly (B).


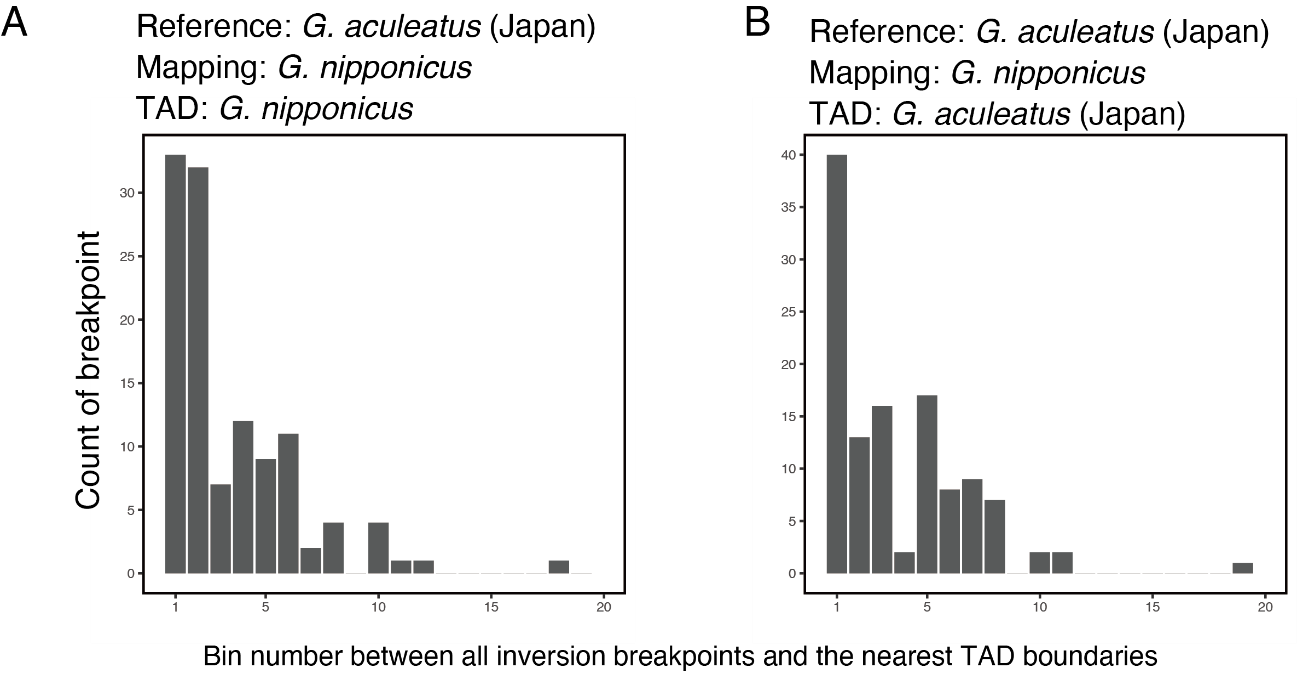


Figure S6

Distance between inversion breakpoints and the nearest TAD boundaries. All inversions between *G. nipponicus* and *G. aculeatus* were used. Distance was measured by the number of bins (25 kbp). TAD boundaries of *G. nipponicus* (A) or *G. aculeatus* (B) were used.


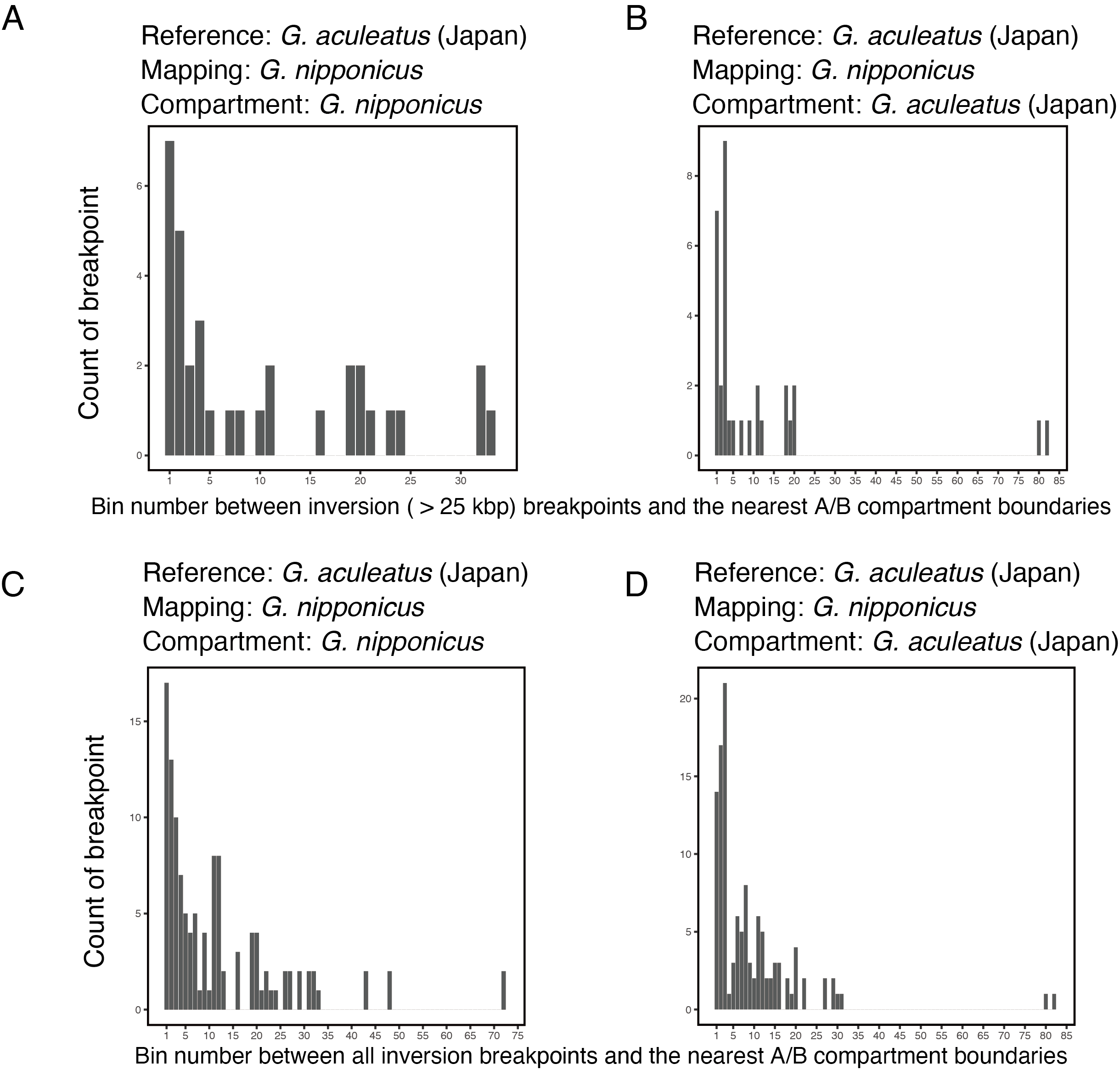


Figure S7

Distance between inversion breakpoints and the nearest A/B compartment boundaries. (A, B) Inversions between *G. nipponicus* and *G. aculeatus* larger than 25 kbp were used. (C, D) All inversions between *G. nipponicus* and *G. aculeatus* were used. Distance was measured by the number of bins (1 bin = 25 kbp).
